# Gingival Blood Flow Velocity by Video Capillaroscopy as an Indicator of Gingival Inflammation

**DOI:** 10.7759/cureus.86837

**Published:** 2025-06-27

**Authors:** Yukie Watanabe, Koji Inagaki, Mari Masuda, Yui Kitamura, Mai Nonaka, Junko Inukai

**Affiliations:** 1 Department of Dental Hygiene, Aichi Gakuin University Junior College, Nagoya, JPN; 2 Department of Periodontology, School of Dentistry, Aichi Gakuin University, Nagoya, JPN

**Keywords:** blood flow velocity, capillaries, gingiva, inflammation, periodontitis, video capillaroscopy

## Abstract

Purpose: We evaluated the usefulness of gingival capillary blood flow velocity (BFV) as an indicator of inflammation in the gingival sulcus.

Methods: Twenty-two non-smoking female dental hygiene students from Aichi Gakuin University Junior College in Nagoya, Japan, with no medication use were assessed for periodontal, gingival blood flow, and nutrient intake. Gingival blood flow was recorded by video capillaroscopy (GOKO-Bscan-ZD). We compared the gingival capillary BFV between sites with and without bleeding on probing (BOP) in the gingival sulcus. For sites with BOP, we further compared BFV based on the presence or absence of visible inflammation.

Results: Nutrient levels did not significantly correlate with BOP or gingival BFV. At the sites with BOP, the gingival capillary BFV (107.3 μm/sec) was significantly slower than that at the sites without BOP (189.3 μm/sec; *P *< 0.001), and the BFV at sites presence of visible inflammation (74.7 μm/sec) was significantly lower than that at sites absent of visible inflammation (142.1 μm/sec; *P *< 0.05).

Conclusion: Gingival capillary BFV is a useful noninvasive indicator that facilitates early quantitative evaluation of localized inflammation within the gingival sulcus.

## Introduction

Gingivitis does not cause major problems initially; however, as it progresses, it can spread to the periodontal tissues and destroy them. Thus, early preventive intervention is crucial [1]. Periodontitis is diagnosed based on periodontal examination [2]. One of the indicators used in this examination is bleeding on probing (BOP), which is a simple and useful marker of inflammation that appears before other gingival findings [3,4]. However, BOP is a qualitative indicator and cannot accurately reflect the severity of inflammation at the examined site. Periodontal tissue has many small blood vessels. In periodontitis or severe gingivitis, vascular dilation and angiogenesis occur that increase capillary density [5,6]. The resultant inflammation alters the structural vascular network, leading to decreased blood flow velocity (BFV) and temporary stagnation of red blood cell transport [7,8].

Studies on gingival microcirculation have used blood flow, oxygen consumption, morphological changes, and capillary density as indicators of the gingival status [7,9-11]. The results of noninvasive laser Doppler blood flowmetry, primarily used for such assessments, are challenging to reproduce, owing to the duration and complexity of the measurements [12,13]. The video capillaroscope device used in this study is known for its high reliability, with excellent reproducibility and diagnostic accuracy of its output, e.g., during the evaluation of nailfold capillaries in systemic sclerosis [14,15]. However, to the best of our knowledge, this instrument has not been used to assess capillary BFV in the human gingiva.

Therefore, in this study, we quantitatively evaluated the degree of gingival inflammation by determining the BFV in gingival capillaries and used BOP as a reference indicator for the diagnosis of periodontitis and other clinical gingival inflammatory findings.

## Materials and methods

Study design and participants

This cross-sectional study included 23 dental hygiene students from Aichi Gakuin University Junior College in Nagoya, Japan, who consented to participate between June 2023 and August 2024. The exclusion criterion was current or former smoking. This was a preliminary study, and a sample size calculation was not performed in advance. 

The data in this study were obtained from the nutritional intake status assessment using a food frequency questionnaire (FFQ) [16] and a questionnaire on smoking status (including heated tobacco products). The FFQ is a reliable data collection tool that estimates energy and nutrient intake from the frequency of habitual food consumption over a specific period [16]. As nutrition plays an important role in periodontal health and microcirculation [17,18], nutritional intake was analyzed using dedicated software (Eiyoplus, Kenpakusha Co., Tokyo, Japan). Periodontal examination included periodontal probing, pocket depth, BOP, assessment of visible inflammatory findings, and gingival evaluation by capillaroscopy. Periodontal examination targeted 36 sites, including the distal, central, and mesial aspects of the buccal side of the anterior teeth in both the maxilla and mandible. Gingivitis was classified based on the etiological factors of gingival inflammation [19], considering systemic conditions, medication status, nutritional intake, and pregnancy. Periodontal examinations were performed, following calibration, by a dental hygienist with clinical experience.

Evaluation of gingival capillaroscopy

**Figure 1:**
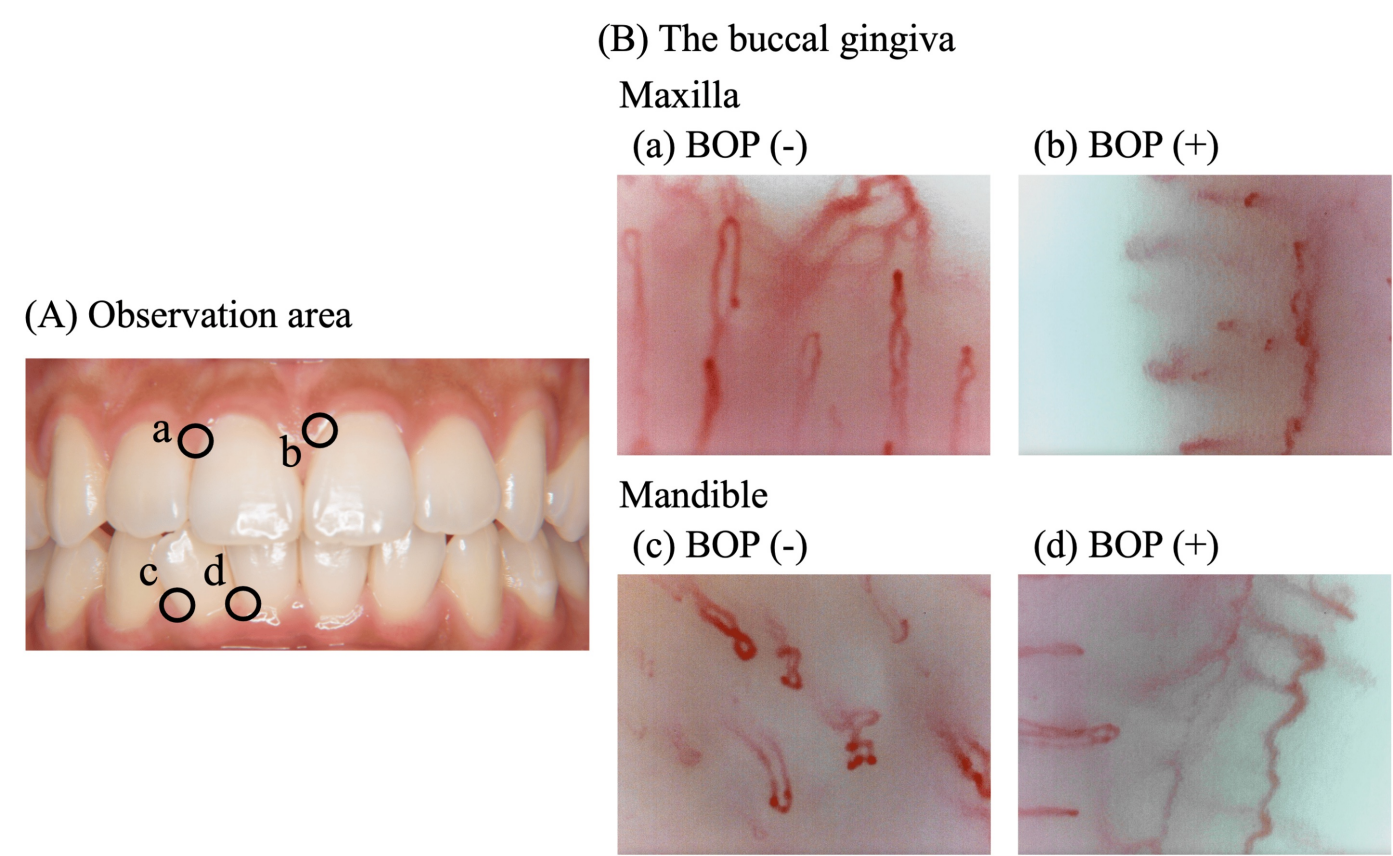
Representative captured images of gingival capillary in subjects with and without bleeding on probing (BOP) The subject was a 22-year-old female. The tooth in question showed an average probing pocket depth of 2.2 mm and bleeding on probing of 16.6%. (A) Gingival capillaries were observed using video capillaroscopy. The gingival blood flow velocities were as follows: (B-a) 826.6 μm/sec, (B-b) 572.2 μm/sec, (B-c) 301.4 μm/sec, (B-d) 237.1 μm/sec.

Capillaroscopy (Bscan-ZD; GOKO Imaging Devices Co., Kanagawa, Japan) was used to observe the gingival capillaries of each participant at approximately ×720 magnification. Measurements were conducted in a room maintained at 23 °C, with participants positioned supine on a dental chair. Blood flow videos were recorded at two sites each in the maxilla and mandible, selected based on the presence or absence of BOP, without specifying tooth type (Figure 1). All four sites were recorded on the same day. To enhance image quality, a colorless and transparent oral moisturizing gel (Butler, Sunstar, Osaka, Japan) was applied to the observation area as an immersion agent. Gingival capillary BFV was analyzed using dedicated software (GOKO-VIP, GOKO Imaging Devices Co., Kanagawa, Japan). The measurement lines were set for three vessels where blood flow was visibly discernible in the recorded videos. The average value for these three vessels was considered as the gingival capillary BFV, and the average of these three sites was used as the BFV for the respective sites. The maxillary and mandibular BFV values were averages of two measurement points. BFV measurements were performed by a dental hygienist who had completed one month of training in equipment use before conducting the periodontal examination.

Statistical analysis

Both BOP and BFV values were non-normally distributed. Spearman's correlation analysis was conducted to evaluate the relationship between BOP and nutrient levels, the association between nutrient levels and BFV, and the relationship between BOP and BFV. Furthermore, the Mann-Whitney *U* test was applied to compare BFV values in the maxilla and mandible based on the site with or without BOP. Data analysis was performed using IBM SPSS Statistics for Windows, Version 28.0 (released 2021, IBM Corp., Armonk, NY). The significance test was two-tailed, and a *P*-value < 0.05 was considered to indicate statistical significance of observed effects.

Ethical considerations

The study was reviewed and approved by the Ethics Committee of Aichi Gakuin University Junior College (Approval No. 23-002). The study followed the guidelines of the Declaration of Helsinki and was conducted based on an informed consent form, which was signed by the patients. All the participants provided informed consent to participate in the study.

## Results

Sample characteristics

As shown in Table 1, of the 23 potential participants, 22 were included in the analysis, and one current smoker was excluded (valid response rate: 95.7%; 100% female, 20 ± 2.4 years). No abnormal nutritional findings were observed. As none of the participants had restorations in the cervical region, were taking medications, or were pregnant, gingival inflammation was attributed solely to bacterial dental biofilms. The average periodontal probing pocket depth was 1.9 mm (standard deviation (SD) = 0.2), and the average BOP was 16.4% (SD = 10.2). Tooth mobility was not observed.

**Table 1:** Sample characteristics The periodontal examination targeted a total of 36 sites, including the distal, central, and mesial aspects of the buccal side of the anterior teeth in both the maxilla and mandible. The blood flow velocity was measured at a total of four sites, with two sites in both the maxilla and mandible for each individual.

	Total (n = 22)	
	Mean	SD
Age (years)	20.9	2.4
Education (years)	14.3	1.2
Height (cm)	157.5	6.0
Weight (kg)	48.8	4.7
Body mass index (kg/m^2^)	19.7	1.8
Nutrition intake		
Energy (kcal)	1,662.6	46.2
Protein (g)	66.4	2.3
Carbohydrates (g)	240.7	7.4
Fat (g)	52.8	3.5
Minerals (g)	7.9	0.4
Vitamins (mg)	135.9	18.8
Number of teeth	27.2	1.5
Periodontal examination		
Probing pocket depth (mm)	1.9	0.2
Bleeding on probing (%)	16.4	10.2
Maxilla (%)	16.2	9.0
Mandible (%)	16.7	12.8
Blood flow velocity	196.7	152.0
Maxilla (μm/sec)	203.2	142.2
Mandible (μm/sec)	193.1	112.7

Relationship between gingival BFV and the site with BOP

**Figure 2:**
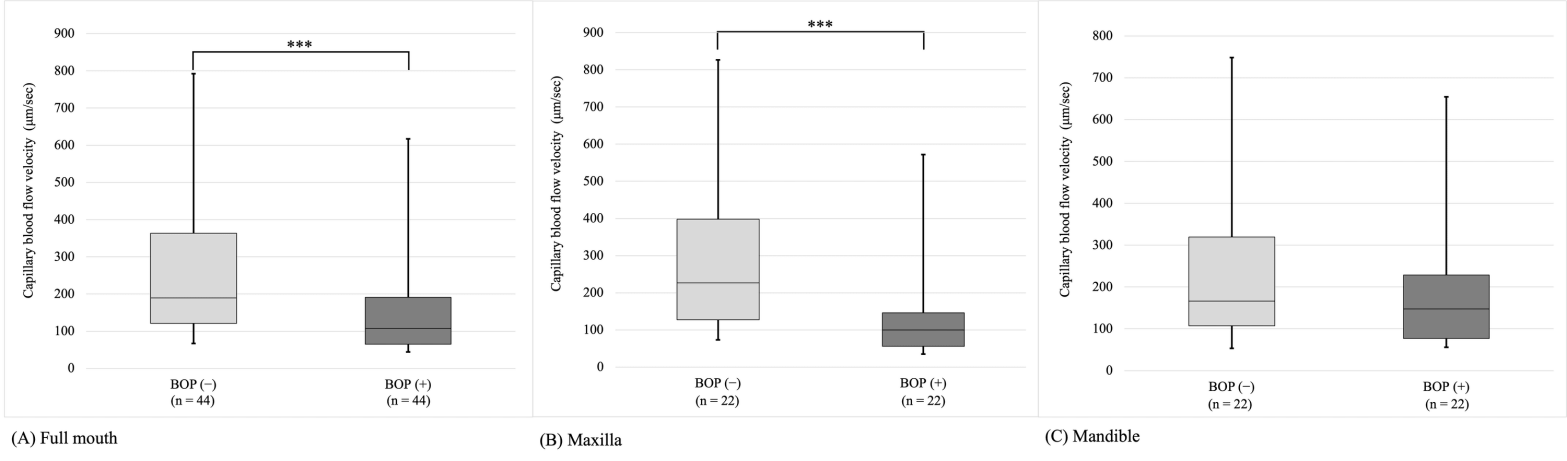
Blood flow velocity at sites with or without bleeding on probing (^***^P < 0.001 in Figure 2) Mann-Whitney U test; BOP: bleeding on probing The associations between gingival blood flow velocity (BFV) and sites with or without bleeding on probing (BOP). (A) BFV values were lower at sites with BOP than at sites without BOP in the full mouth. (B) BFV values were also lower at sites with BOP than at sites without BOP in the maxilla. (C) There was no significant difference in the mandible.

Nutrient levels were not significantly associated with BOP and BFV (Table 2). Therefore, we did not consider nutritional confounding between BFV and inflammation. Table 3 and Figure 2 present the BFV results. Full mouth and maxillary BFV values were lower at the sites with BOP than at the sites without BOP (*P* < 0.001 in both cases). By contrast, mandibular BFV did not differ between the sites with and without BOP.

**Table 2:** Correlation between nutrient intake and bleeding on probing score and blood flow velocity

	Bleeding on probing			Blood flow velocity (full mouth)		
	Correlation coefficient (r)	*P*-value	95% confidence interval for r	Correlation coefficient (r)	*P*-value	95% confidence interval for r
Energy (kcal)	-0.07	0.756	-0.488–0.373	-0.027	0.907	-0.558–0.405
Protein (g)	-0.061	0.788	-0.481–0.382	-0.005	0.982	-0.545–0.459
Carbohydrates (g)	-0.082	0.718	-0.497–0.364	0.135	0.549	-0.348–0.526
Fat (g)	0.042	0.853	-0.398–0.466	-0.030	0.895	-0.578–0.458
Minerals (g)	-0.132	0.557	-0.534–0.318	-0.004	0.986	-0.498–0.455
Vitamins (mg)	-0.129	0.568	-0.532–0.322	-0.125	0.580	-0.534–0.314

**Table 3:** Blood flow velocity at sites with or without BOP Mann-Whitney U test; BOP: bleeding on probing

	n	BOP (-)	n	BOP (+)	*P*-value
		Median (IQR)		Median (IQR)	
Full mouth	44	189.3 (120.9-363.7)	44	107.3 (65.2-191.3)	<0.001
Maxilla	22	226.8 (127.5-398.0)	22	99.7 (55.8-146.3)	<0.001
Mandible	22	166.0 (107.3-319.6)	22	147.4 (76.9-228.7)	0.197

Relationship between periodontal parameters and gingival capillary BFV

No correlation was observed between BOP score and gingival BFV.

Association of visible inflammatory findings and gingival BFV in the sites with BOP

**Figure 3:**
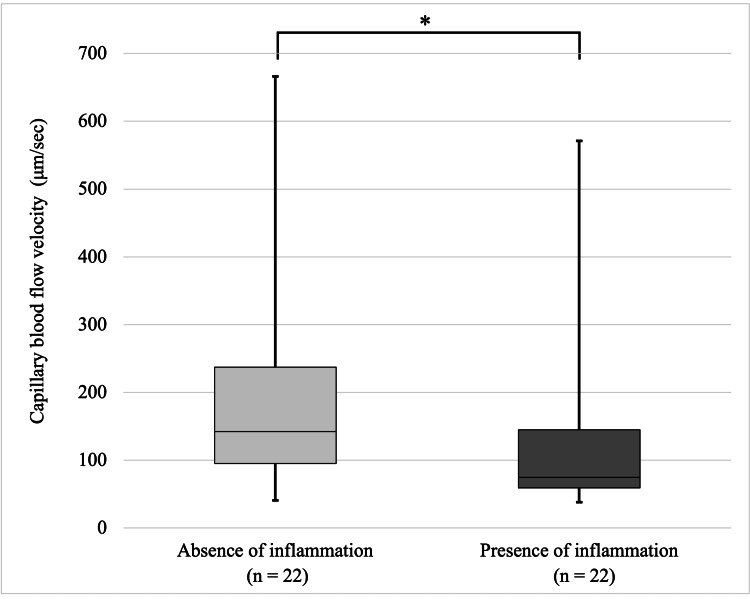
Blood flow velocity by visible inflammatory findings in sites with bleeding on probing (^*^P < 0.05 in Figure 3) Mann-Whitney U test The associations between clinical inflammatory findings and gingival blood flow velocity (BFV) in the site with bleeding on probing (BOP). BFV values was slower in areas with inflammatory findings  than in those absent of inflammation.

Gingival BFV was slower in areas with inflammatory findings (74.7 μm/sec) than in those absent of inflammation (142.1 μm/sec) (*P* < 0.05) (Figure 3).

## Discussion

In previous studies that utilized a video capillaroscope, indices such as capillary density, changes in capillary morphology, and capillary length were determined [14,20-23]. However, very few studies have explored BFV [8,24-26], and this report is the first investigation of BFV in the oral cavity undertaken using video capillaroscopy, which indicates the considerable importance of this study. 

No specific tooth sites were identified. Instead, the sites where BOP was observed on the measurement day were selected as observation targets, and the BFV values were compared based on sites with or without BOP. In addition, absences of medication use and pregnancy were confirmed, and gingival inflammation was determined to be plaque-induced before the analysis. When activated by inflammatory mediators, chronic inflammation increases the permeability of endothelial cells and promotes the adhesion and migration of white blood cells. This changes the structure and function of blood vessels and contributes to vascular narrowing and stagnation of blood flow, which significantly affects the progression of diseases associated with chronic inflammation [27,28]. Plaque-induced gingivitis, a type of chronic inflammation, similarly activates the immune-inflammatory response owing to the accumulation of dental plaque, leading to histological changes such as increased vascular permeability and collagen degradation [19]. Consequently, changes in local microcirculation occur, including reduced blood flow and alterations in vascular structure [29]. This study revealed that the BFV was slower at the sites with BOP. Previous studies have suggested that gingivitis caused by plaques leads to the adhesion of white blood cells, formation of blood clots, and obstruction of blood flow, resulting in changes in the hemodynamics within the gingival microcirculation, which in turn slows BFV. However, BFV in the superficial capillaries of the gingiva remained unchanged despite the inflammatory state [8]. However, a statistically significant difference in our study likely resulted from a larger sample size. 

In the present study, significant differences were observed only in the maxilla, but not in the mandible. A likely reason for this is that blood flow in the gingiva of the mandibular anterior region is reportedly lower than that in the maxillary anterior region [30]. Consequently, the baseline gingival BFV in the absence of BOP, which indicates the absence of gingival inflammation, may also be lower in the mandible than in the maxilla. This could explain the lack of differences observed between the sites with and without BOP in the mandible. In addition, as BOP values did not correlate with BFV, the significant difference in BFV between the sites with or without BOP in both the maxilla and mandible suggests that BFV could be a useful indicator for quantitatively evaluating the effects of inflammation on the local gingival microcirculation. In addition, as the study included only 22 participants, it is clear that further accumulation of data is necessary to establish the baseline gingival BFV for both the maxilla and mandible.

The video capillaroscopy instrument used in this study visualizes capillaries on the body surface. Therefore, it does not directly monitor inflammation within the periodontal pockets. However, a difference in BFV was observed between the sites with BOP, which is an earlier sign of inflammation than gingival color changes [4], and those with or without macroscopic inflammation. This suggests that BFV could be an effective tool for assessing the degree of inflammation before the latter becomes visible on the gingival surface, supporting a previous claim [4].

This study had four notable limitations. First, all participants in this study were female, and factors such as the menstrual cycle may have influenced the results. However, these potential effects were not considered in our analyses. Second, although gingival inflammation is reportedly associated with nutritional intake, no such correlation was observed in this study. Consequently, the relationship between nutrition and gingival inflammation was not examined further. Future studies should use larger sample sizes to further explore the potential association between nutritional status and BFV. Third, because this study targeted only Japanese women, it is difficult to generalize the findings to other populations. Finally, the absence of significant associations between nutritional status and BOP or BFV may be due to the small sample size and resulting limited statistical power. The homogeneity of the healthy female participants, with minimal variation in diet and lifestyle, may have also influenced the results. Future studies should include a wider age range to explore these associations further. However, the potential capacity for noninvasive evaluation of inflammation within the periodontal pocket from the surface provides significant value in the quantification of the degree of gingival inflammation. The BFV of gingival capillaries potentially constitutes a noninvasive and quantitative marker for the evaluation of inflammation in the gingival sulcus before it becomes visible to the naked eye.

## Conclusions

This study provides a new perspective on the assessment of gingivitis. Traditionally, the evaluation of gingival inflammation has relied on visual inspection and probing for BOP. However, our findings suggest that changes in gingival capillary blood flow may occur even before the appearance of BOP, potentially enabling earlier detection of inflammation. Notably, video capillaroscopy is a non-invasive technique, making it a promising tool for the objective and quantitative assessment of gingivitis.

With further advancements in video capillaroscopy technology and a clearer path toward clinical application, this method could become a valuable tool for the prevention, diagnosis, and monitoring of periodontal disease. In particular, establishing a technology that allows real-time evaluation of periodontal tissue health could contribute to improved diagnostic accuracy in dental clinical practice.
